# Including service users in trials and rigorous studies in health and social care: developing a standard operating procedure for researchers

**DOI:** 10.1186/1745-6215-12-S1-A83

**Published:** 2011-12-13

**Authors:** Bridie Angela Evans, Emma Bedson, Philip Bell, Hayley Hutchings, Lesley Lowes, David Rea, Anne Seagrove, Stefan Siebert, Graham Smith, Helen Snooks, Marie Thomas, Kym Thorne, Ian Russell

**Affiliations:** 1 College of Medicine, Swansea University, Swansea, SA2 8PP, UK; 2 North Wales Organisation for Randomised Trials in Health (NWORTH), Bangor University, Bangor, LL57 2DG, UK; 3 Service User, Cardiff University, CF14 4XN, UK; 4 School of Nursing and Midwifery Studies, Cardiff University, CF14 4XN, UK; 5 College of Human and Health Sciences, Swansea University, Swansea, SA2 8PP, UK; 6 School of Education, University of Wales Newport, Newport, NP18 3QT, UK

## Background

Involving service users in research is encouraged as a way to improve research quality, relevance and accountability and is a pre-requisite for many funding bodies. Existing guidance for researchers on how to do this mainly discusses general principles. Some researchers may question the value, feasibility and impact of including service users and limit the scope of involvement. We defined service users as patients, carers, people eligible for a service or anyone relevant to the trial inclusion criteria.

## Objective

To develop a standard operating procedure (SOP) to give researchers detailed guidance on how to include service users in trials and other rigorous studies.

## Methods

Researchers with experience of service user inclusion and service users currently involved in trials adopted by the West Wales Organisation for Rigorous Trials in Health (WWORTH), the Clinical Trials Unit based in Swansea University, collaborated to develop the SOP for service user inclusion.

## Results

We articulated core principles of equality underpinning the SOP and guidance on how to achieve these. Processes to recruit and engage service users were set out. We developed a framework for inclusion in research which defined minimum core levels of collaboration plus additional consultation and collaboration opportunities. A flow diagram identified when and how to include service users at each of the Medical Research Council’s five stages of developing and evaluating complex interventions in health [1]. We listed people across the research team responsible for including service users in studies and promoting an inclusion culture and highlighted importance of training for researchers and service users. We stated that service users should be included as early as possible in the research process with a minimum of two on all formal trial groups and committees. We proposed a minimum 1% of total research budget should be set aside to include service users and sufficient additional time built in to allow for full inclusion.

## Conclusion

Supporting good practice when including service users in research could benefit the relevance, accountability and quality of health and social care research. This SOP provides guidance to researchers to successfully involve service users in developing proposals, undertaking rigorous research and creating a culture of routine service user inclusion in research at all stages. The UK Clinical Research Collaboration should require trials to demonstrate service user inclusion and research funders must set aside sufficient funds and time for this in research proposals.
